# Clinical management outcomes of childhood glaucoma suspects

**DOI:** 10.1371/journal.pone.0185546

**Published:** 2017-09-25

**Authors:** Matthew B. Greenberg, Carla J. Osigian, Kara M. Cavuoto, Ta C. Chang

**Affiliations:** 1 University of Miami Miller School of Medicine, Miami, Florida, United States of America; 2 Bascom Palmer Eye Institute, Miami, Florida, United States of America; Duke University, UNITED STATES

## Abstract

**Purpose:**

To investigate the outcomes of childhood glaucoma suspects.

**Design:**

Retrospective case series.

**Methods:**

Records of childhood glaucoma suspects were identified using financial claims data; medical history, baseline biometric and exam findings were recorded. Conversion from suspect to glaucoma was determined based on the Childhood Glaucoma Research Network criteria. The study adheres to the tenets of the Declarations of Helsinki.

**Results:**

214 subjects were enrolled, with median age at initial presentation of 6.37 years (interquartertile range: Q1 = 2.46, Q3 = 8.90). 22 (10.2%) subjects developed glaucoma, 64 (29.9%) had ocular hypertension but no glaucoma, 9 (4.2%) had high-risk condition or syndrome without either ocular hypertension or glaucoma after a mean follow up of 39 +/- 34 months. Neither a family history of glaucoma nor patient gender was significantly different between the groups. 40.2% of subjects (86 of 214) had two or more episodes of intraocular pressure (IOP) > 21 mmHg, among which 25.6% (22 of 86) developed glaucoma after a mean duration of 32.8 +/- 33.5 months.

**Conclusions:**

Up to 25% of children with 2 or more episodes of elevated IOP may develop glaucoma. In 50% of suspects who converted to glaucoma, elevated IOP was not present at the initial evaluation. There is no significant difference in gender, family history, or baseline central corneal thickness between suspects who developed glaucoma compared to the rest. While suspects who converted to glaucoma had higher average, maximum and minimum IOP measurements, there is no clear cutoff between the groups.

## Introduction

Childhood glaucoma is a heterogeneous group of diseases that result in pressure-related damage to the developing ocular structures [1], and accounts for 2–6% of blindness in children world-wide [2]. In 2013, the Ninth World Glaucoma Association Consensus in conjunction with the Childhood Glaucoma Research Network (CGRN) provided formal definitions for childhood glaucoma as well as childhood glaucoma suspect [1]. In clinical practice, pediatric patients are considered glaucoma suspects if they have elevated intraocular pressure (IOP), optic disc appearance or visual field findings suggestive of glaucoma, or anterior segment and/or biometric findings suspicious for glaucoma without meeting the diagnostic criteria for childhood glaucoma. In addition, children may be referred for glaucoma evaluation based on the presence of a high-risk ocular findings, condition or systemic syndrome.

Overall, childhood glaucoma suspects constitute a significant proportion (approximately 38.5%) of the patients seen in a referral pediatric glaucoma service [3]. The incidence and risk factors of glaucoma conversion in these childhood glaucoma suspects are unknown. In this retrospective study, we investigated the baseline demographic and ocular characteristics as well as the outcomes of childhood glaucoma suspects managed in a tertiary referral center.

## Materials and methods

### Study population

A protocol for retrospective review of medical records was approved by the Institutional Review Board (IRB) of the University of Miami. All data were extracted in an anonymized, de-identified fashion and aggregated prior to analysis; the data collection method was reviewed and approved by the IRB. Potential subjects were identified using a financial claims search for International Classification of Diseases 9^th^ and 10^th^ Editions (ICD-9/ICD-10) codes for “glaucoma suspect” or “ocular hypertension” for visits between 2002 and 2012. The subjects were enrolled if they were <18 years of age at the time of the first clinic visit, and if a diagnosis of “ocular hypertension” and/or “glaucoma suspect” was documented in the clinic note. They were excluded if they: 1) met the CGRN criteria for the diagnosis of childhood glaucoma upon initial evaluation, 2) had a history of prior intraocular surgery at the time of the initial visit, 3) had comorbidity that prevents accurate assessment of IOP (e.g. corneal abnormalities), or 4) had less than 6 months of follow up. Baseline patient demographic information and examination findings from each follow up clinic visit were recorded. Baseline automated visual field (Humphrey Field Analyzer, Carl Zeiss Meditec, Dublin, CA; 30–2 or 24–2 protocol, any stimulus size; SITA-standard, SITA-fast or FastPac programs), when available, was graded as “normal,” “abnormal but not glaucomatous,” and “glaucomatous” by a fellowship-trained glaucoma specialist (TCC). If both eyes of a patient were eligible for study, the eye with higher IOP on initial evaluation was included. The decision to initiate treatment was at the discretion of the attending pediatric glaucoma specialist; no criteria or protocol were used in this decision-making.

### Definitions

Patients were divided into four groups based on the diagnosis of their last visit: Group 1 –glaucoma, Group 2 –ocular hypertension, Group 3 –high-risk condition or syndrome, and Group 4 –other. Group 2 included any subject who had IOP > 21 mmHg on two or more occasions but did not meet the CGRN criteria for glaucoma diagnosis at the end of the follow up period. Any subject with an ocular condition or systemic syndrome associated with an increased risk of developing childhood glaucoma [1] was categorized into Group 2 if they have demonstrated IOP > 21 mmHg on two or more occasions, and was categorized into Group 3 if they had not demonstrated elevated IOP throughout the study period. Subjects without glaucoma, high-risk condition/syndrome or ocular hypertension through the study period who were followed as a glaucoma suspect for other reasons (e.g. family history, increased cup/disc ratio [CDR]) were categorized into Group 4. Conversion from being a glaucoma suspect to manifest glaucoma is declared if two or more of the following findings are present:

1. A progressive increase in cup-disc ratio or focal rim thinning as documented on serial disc photos not attributed to non-glaucomatous causes;
2. A progressive thinning of circumpapillary retinal nerve fiber layer (RNFL) of ≥ 10 microns that is not attributed to image acquisition artifacts and non-glaucomatous causes;
3. Progressive myopic shift coupled with an increase in ocular dimensions out of keeping with normal growth, in the context of elevated IOP > 21 mmHg on two or more occasions;
4. An acquired visual field defect, or a reproducible deepening and/or expansion of a pre-existing visual field defect that is consistent with glaucomatous optic neuropathy that is not attributed to non-glaucomatous causes.

### Statistical analysis

The baseline demographic and ocular characteristic between the four groups were compared and analyzed. Categorical variables were compared using Chi-square test, while quantitative variables were compared using one-way analysis of variance. A p-value ≤ 0.05 was considered significant.

## Results

The initial ICD-9/ICD-10 search resulted in 546 entries, of which 214 subjects met the inclusion and exclusion criteria and were enrolled. The median age at initial presentation was 6.37 years (interquartertile range: Q1 = 2.46, Q3 = 8.90) and a mean (+/- standard deviation [SD]) follow up duration of 39 +/- 34 months. At the end of the follow up period, 22 (10.2%) subjects were in Group 1 (glaucoma) based on the CGRN criteria (4 late-onset primary congenital glaucoma, 4 associated with ocular inflammation, 4 juvenile open angle glaucoma; 3 associated with port-wine birthmark, 3 following trauma, one associated with Marfan syndrome, one associated with Axenfeld-Rieger syndrome, one associated with congenital persistent fetal vasculature without prior surgery, and one associated with oculodentodigital dysplasia), 64 (29.9%) were in Group 2 (ocular hypertension), 9 (4.2%) were in Group 3 (high-risk condition or syndrome), and 119 (55.6%) were in Group 4 (other). The follow up duration of each group was not significantly different (mean for Groups 1, 2, 3 and 4 were 32.8 +/- 33.5 months, 43.8 +/- 37.4 months, 33.1 +/- 26.6 months, and 37.7 +/- 32.3 months, respectively; p > 0.10). Neither a family history of glaucoma (Χ^2^ = 2.7, p = 0.43) nor patient gender (p = 0.40) were significantly different between the groups. Altogether, 40.2% (86 of 214) subjects had two or more episodes of IOP > 21 mmHg. Of these patients, 25.6% (22 of 86) developed glaucoma after a mean follow up of 32.8 +/- 33.5 months. Patient demographics and baseline characteristics are summarized in Table 1. The diagnoses in Group 3 (high-risk condition or syndrome) in this cohort included: uveitis, trauma, Rubinstein-Taybi syndrome, aniridia, microphthalmia, Axenfeld-Rieger syndrome and neurofibromatosis type I.

**Table 1:** Patient demographics and baseline characteristics.

	Group 1	Group 2	Group 3	Group 4	P-value**
N (%)	22 (10.2)	64 (29.9)	9 (4.2)	119 (55.6)	
Age (years)*	5.16 +/- 4.17	6.24 +/- 3.62	2.37 +/- 3.06	6.40 +/- 3.69	0.01
Follow up duration (months)*	32.8 +/- 33.5	43.8 +/- 37.4	33.1 +/- 26.6	37.7 +/- 32.3	0.48
Male gender	40.9%	53.1%	33.3%	52.9%	0.40
Positive family history	13.6%	28.1%	11.1%	24.4%	0.43
Average IOP*	25.5 +/- 7.15	20.3 +/- 2.98	16.6 +/- 3.57	15.9 +/- 2.23	<0.01
Maximum IOP*	34.3 +/- 7.23	26.1 +/- 3.81	17.8 +/- 1.09	18.0 +/- 2.31	<0.01
Minimum IOP*	18.3 +/- 8.50	17.1 +/- 3.86	14.8 +/- 4.27	14.1 +/- 3.04	<0.01
Baseline axial length*	22.9 +/- 4.03	20.2 +/- 1.44	21.8 +/- 0.42	22.4 +/- 1.41	0.07
Baseline CDR*	0.55 +/- 0.25	0.52 +/- 0.21	0.39 +/- 0.20	0.57 +/- 0.15	0.02
Baseline RNFL thickness*	87.6 +/- 21.41	95.5 +/- 14.57	95.0 +/- 14.14	101.3 +/- 11.79	0.02
Baseline corneal diameter*	11.16 +/- 1.02	11.04 +/- 1.25	10.60 +/- 0.96	11.424 +/- 1.05	0.68
Baseline CCT	601.8 +/- 125.91	574.5 +/- 37.56	545***	563.3 +/- 42.00	0.23

Group 1 –glaucoma; Group 2 –ocular hypertension; Group 3 high-risk condition or syndrome; Group 4 –other; CDR = cup-disc ratio; CCT = central corneal thickness;

*mean +/- standard deviation;

**one-way analysis of variance;

***only one subject in this group had CCT measured at baseline

On initial evaluation, 50% of suspects who eventually developed glaucoma (Group 1) had baseline IOP < 21 mmHg, with elevated IOP noted on follow up visits. Group 1 had the highest average, maximum and minimum IOP (25.5 +/- 7.1, p<0.01, 34.3 +/- 7.2, p<0.01, and 18.3 +/- 8.5, p<0.01, respectively), and displayed an insignificant trend towards a higher baseline axial length (mean 22.9 +/- 4.0, p = 0.07). Group 3 (high-risk conditions or syndromes without elevated IOP) had a significantly lower baseline cup-disc ratio (CDR; mean 0.39 +/- 0.19, p = 0.02). Group 4 (other) had significantly higher baseline mean circumpapillary RNFL thicknesses (mean 101.3 +/- 11.8, p = 0.02), while Group 1 had the lowest mean thicknesses (mean 87.6 +/- 21.4). There was not a significant difference in baseline visual field findings (Χ^2^ = 9.7, p = 0.5). In addition, no difference was seen in baseline corneal diameters or central corneal thickness (CCT; p >0.10 for all).

## Discussion

Although childhood glaucoma suspects constitute the largest proportion of patients seen in a referral pediatric glaucoma service [3], little is known about the clinical course of these patients. To the authors’ knowledge, this is the first study to investigate the incidence and risk factors of glaucoma conversion in childhood glaucoma suspects. Approximately half of our cohort (55.6%) did not demonstrate more than one episode of elevated IOP and did not have high-risk conditions or syndromes for childhood glaucoma. Of the 44.4% of the patients who demonstrated two or more episodes of elevated IOP during the study period, 25.6% converted to glaucoma after a mean follow up of approximately 2.8 years. This suggests that a significant portion of childhood glaucoma suspects, especially those with two or more episodes of IOP > 21 mmHg, do go on to develop glaucoma, and that structural and functional stability on short-term follow up does not rule out the possibility of future glaucoma conversion. Furthermore, since only 50% of those who developed glaucoma presented with baseline IOP > 21 mmHg, the absence of elevated IOP on initial evaluation does not obviate the need for long-term monitoring.

At the final visit, the subjects with high-risk condition or syndrome but remained without either glaucoma or elevated IOP (Group 3) were significantly younger than the other groups. This is likely an artifact of categorization, as any cohort member who acquired elevated IOP and/or glaucoma over the study period was reassigned to either Group 1 or Group 2. A prior survey showed that glaucoma associated with nonacquired systemic disease, syndrome or ocular anomalies present with a median age of between 0.5 and 2.9 years [3], which suggested that older children with high-risk conditions or syndromes are less likely to remain normotensive or without glaucoma and thus remain in Group 3.

While the average, maximum and minimum IOP were highest in patients who converted to glaucoma, there were no distinct cutoffs that separated the group that converted to glaucoma from those who remained suspects. We found higher baseline RNFL in normotensive suspects without high-risk conditions or syndromes (Group 4) compared to subjects who developed glaucoma (Group1), which suggests that a thin baseline average RNFL measurement may be associated with an increased risk of glaucoma development in the pediatric cohort.

The lack of difference in corneal diameter among the four groups was not surprising, as any child with obvious buphthalmos would likely have met the criteria for childhood glaucoma on the initial evaluation and have been excluded. However, the lack of difference in central corneal thickness between the groups is in contrast to prior studies, which showed a significantly increased baseline CCT in glaucoma patients [4–5]. Our finding may be attributed to the fact that many of our patients with ocular hypertension but no glaucoma (Group 2) and high-risk syndromes without glaucoma (Group 3) may have conditions associated with increased CCT, such as microcornea and aniridia [6–8]. Since the role of CCT in childhood tonometry remains uncertain [1], and adjustment of IOP based on central corneal thickness does not improve prediction models for primary open angle glaucoma in adults [9], it would be inappropriate to adjust IOP in childhood glaucoma suspects based on pachymetry.

This study has several imitations. The retrospective design in a tertiary care setting may introduce selection bias. Childhood glaucoma suspects who are thought to have a very low risk of glaucomatous conversion, i.e. a child referred for a family history of adult-onset glaucoma, may have been followed elsewhere after an initial consultation at our institution, and was thus excluded from the analyses. This would concentrate the pool of high-risk glaucoma suspects in our cohort and thus yield a higher conversion rate. On the other hand, the initial medical record search was designed to catch those diagnosed with “ocular hypertension” or “glaucoma suspect,” which may have missed patients with high-risk conditions or syndromes (e.g. chronic steroid usage, Lowe syndrome, etc.) but were not coded as suspects. Our exclusion of Peters anomaly (due to the presence of corneal abnormalities and inability to obtain accurate IOP measurements) and patients with prior intraocular surgery may limit the generalizability of our findings. Our protocol precluded subgroup analyses of glaucoma conversion risk based on initial categorization, which may further limit the data’s application in prognosticating glaucoma in high-risk children.

In summary, childhood glaucoma suspects require periodic monitoring, with special vigilance paid to those with two or more episodes of elevated IOP, as up to 25% may convert to glaucoma. The potential benefit of initiating prophylactic IOP-lowering therapy should be weighed against the risk and burden of topical therapy, especially in young children with minimally elevated IOP and no other risk factors. Since 50% of suspects who eventually converted to glaucoma did not present with elevated IOP, normal IOP on presentation does not preclude the need for monitoring. There was no significant difference in gender, family history, or baseline CCT between suspects who developed glaucoma compared to those who did not. While suspects who converted to glaucoma had higher average, maximum and minimum IOP measurements, there was no clear cutoff between the groups. Future efforts should focus on elucidating the amount of risk reduction per increment of IOP reduction in pediatric ocular hypertension patients.
